# Point Mutation Specific Antibodies in B-Cell and T-Cell Lymphomas and Leukemias: Targeting IDH2, KRAS, BRAF and Other Biomarkers RHOA, IRF8, MYD88, ID3, NRAS, SF3B1 and EZH2

**DOI:** 10.3390/diagnostics11040600

**Published:** 2021-03-27

**Authors:** Kunwar Singh, Sumanth Gollapudi, Sasha Mittal, Corinn Small, Jyoti Kumar, Robert S. Ohgami

**Affiliations:** 1 Department of Pathology, Stanford University, Stanford, CA 94063, USA; kumarj@stanford.edu; 2 Department of Pathology, University of California, San Francisco, CA 94143, USA; Sumanth.Gollapudi@ucsf.edu (S.G.); sashamittal11@gmail.com (S.M.); Corinn.Small@ucsf.edu (C.S.)

**Keywords:** single nucleotide polymorphisms, hematopathology, cancer, diagnostics, point mutation specific antibodies, precision medicine

## Abstract

B-cell and T-cell lymphomas and leukemias often have distinct genetic mutations that are diagnostically defining or prognostically significant. A subset of these mutations consists of specific point mutations, which can be evaluated using genetic sequencing approaches or point mutation specific antibodies. Here, we describe genes harboring point mutations relevant to B-cell and T-cell malignancies and discuss the current availability of these targeted point mutation specific antibodies. We also evaluate the possibility of generating novel antibodies against known point mutations by computationally assessing for chemical and structural features as well as epitope antigenicity of these targets. Our results not only summarize several genetic mutations and identify existing point mutation specific antibodies relevant to hematologic malignancies, but also reveal potential underdeveloped targets which merit further study.

## 1. Introduction

**Figure 1:**
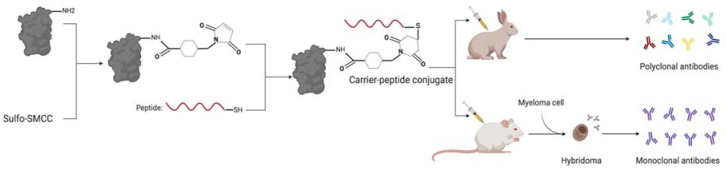
Example of a conventional targeted monoclonal and polyclonal antibody development process using conjugated peptides.

In 2016, the World Health Organization (WHO) revised the fourth edition of the *WHO Classification of Tumours of Haematopoietic and Lymphoid Tissues* based on new molecular and morphological findings [1]. The revised criteria for numerous hematopoietic malignancies indicate that genetic mutations play a significant role in the diagnosis of these entities. Many of these mutations are well-defined recurrent point mutations in specific genes. While such mutations can be detected using genetic sequencing methodologies, another strategy for identification is to develop antibodies that specifically target mutated sites in proteins present within patient tissue samples [2,3]. Monoclonal or polyclonal antibodies can also be developed against these mutations by conjugating the relevant peptide to a carrier protein and injecting the peptide-protein complex into a host animal (Figure 1).

Many biotechnology companies produce antibodies that target commonly expressed proteins but there is a paucity of antibodies available on the market that are designed to target point mutations related to T-cell and B-cell malignancies. The ability to use antibodies in hematologic malignancies would provide benefit both in research and clinical settings. Compared to next-generation sequencing (NGS) assays, in which results and interpretation can take weeks to finalize, in situ immunohistochemistry (IHC) assays are a sensitive and often more efficient approach. In order to expedite the diagnosis and treatment of hematologic malignancies, a tiered system consisting of early IHC and follow up NGS is used in many clinical settings and has been proposed in some published studies [4]. However, a limitation to this rapid protocol, in certain settings, is the availability of mutation-specific antibodies. 

In this study, we aim to describe both current and potential biomarker targets in T-cell and B-cell neoplasms, highlighting several point mutations for their suitability in antibody development based on their predicted antigenicity in relation to multiple chemical and structural parameters.

## 2. Materials and Methods

### 2.1. Target Selection

For our study, we selected genes with known point mutations that have been described in hematolymphoid neoplasms that occur at a minimum frequency of 10% within each diagnostic entity. We also included mutations that influence diagnostic or prognostic subtyping based on WHO criteria.

Antibodies for point mutations available on market or shared freely from research groups were identified using Biocompare (https://www.biocompare.com/, accessed on 4 January 2021), web-based search engine queries, and major antibody vendor websites (i.e., Millipore Sigma, BioLegend, and Abcam).

### 2.2. Peptide and Antibody Evaluation 

The potential for point mutation specific antibody production of the selected genes was assessed by using peptide sequences from the associated proteins that contained the mutated amino acids, which were identified using NCBI’s GenBank and UniProt. The sequences used for analysis consisted of the targeted point mutation site, the 10 amino acids upstream and the 10 amino acids downstream of the target, resulting in a 21-mer for analysis. Solubility was determined using the PepCalc calculator (https://pepcalc.com/, accessed on 28 December 2020) by Innovagen AB [5]. Three crosslinkers were assessed for each peptide regarding appropriate conjugation chemistry to carrier proteins (KLH or BSA); (1) m-Maleimidobenzoyl-N-hydroxysuccinimide ester (MBS), (2) 1-ethyl-3-[3-dimethylaminopropyl]carbodiimide hydrochloride (EDC), and (3) activated EDC (aEDC). 

A main factor considered for crosslinker selection was the ability to bind to residues without reacting to nearby side chains, which could lead to incorrect conjugation to the carrier protein. MBS is a crosslinker compound with a maleimide reactive group at one end and an ester group on the other end. The maleimide group reacts with sulfhydryl groups to form thioester linkages, therefore the 21-mer should not contain cysteine residues. EDC is a carbodiimide, and its reactivity to carboxyl groups leads to direct conjugation to a peptide’s free amine group. As a result, the side chains of aspartic and glutamic acid residues, both of which contain carboxylate ions, can react with EDC. Finally, aEDC interaction with carboxylate groups forms N-hydroxysuccinimide (NHS) esters, which conjugate to amine groups. To avoid errant conjugation, the 21-mer should not have a lysine residue, as its side chain contains a protonated amine group that will not be sterically hindered from binding.

Sequence complexity was computed using the Simple Modular Architecture Research Tool (SMART) (http://smart.embl-heidelberg.de/, accessed on 28 December 2020) [6]. The sequence readout identifies evolutionarily conserved protein domains as well as amino acid sequences with low compositional complexity. SMART uses the SEG algorithm to determine low sequence complexity based on a measure that accounts for potentially biased residue compositions [7]. Surface epitope exposure was estimated using the POLYVIEW-2D structure visualization server (https://polyview.cchmc.org/, accessed on 24 January 2021) [8]. Changes in relative solvent accessibility (RSA) ranging from 0 (completely buried) to 9 (fully exposed) along with physical and chemical properties (hydrophobicity, polarity, and charge) identified interaction interfaces along the peptide. These interaction interfaces are essential for predicting antibody-native protein reactivity. To use the server, the four-character PDB code was inputted and set to be considered as an asymmetric unit. The structural data from predictions was queried, and the numerical solvent accessibility information was selected. All remaining standard criteria were retained, and the first result was used for generating the predictions. The values for each mutation are recorded in Table 1. 

## 3. Results

We identified eleven targets for this study and summarize findings below, detailing the molecular biology relevant to the target, how the mutation disrupts normal biological pathways, and the significance of the genetic abnormality in hematopoietic neoplasms (Table 1). 

### 3.1. BRAF V600E

BRAF is a serine/threonine protein kinase activating the mitogen-activated protein (MAP) kinase/extracellular signal regulated (ERK) signaling pathway which plays a role in cellular division, differentiation, and survival. Typically, Raf activations are downstream targets of the RAS pathway and stimulate a signaling cascade by phosphorylation of MAPK which successively phosphorylates and activates downstream proteins. Most of the mutations found in this gene occur in exon 15 which results in a valine to glutamic acid substitution at codon 600 (i.e., V600E) and is thought to mimic phosphorylation of the activation site [20]. In turn, this alteration acts as a proto-oncogene causing constitutive activation.

**Figure 2:**
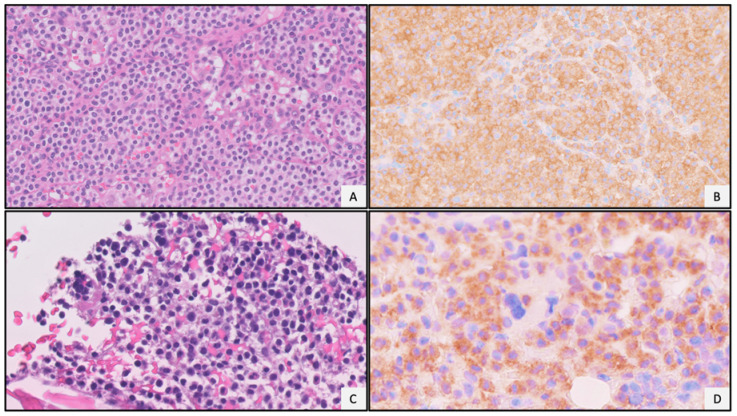
Examples of *BRAF* V600E point mutation specific antibody in a case of hairy cell leukemia. (**A**) Hematoxylin and eosin (H&E) stain of lymph node demonstrates diffuse monotonous proliferation of atypical lymphocytes with a characteristic “fried-egg” appearance, representing nodal involvement by hairy cell leukemia (magnification ×20). (**B**) *BRAF* V600E point mutation specific antibody shows diffuse cytoplasmic expression within the lymphomatous infiltrate (magnification ×20). (**C**) Atypical lymphomatous infiltrate within the bone marrow space (magnification ×20, H&E stain). (**D**) *BRAF* V600E immunohistochemical stain showing diffuse cytoplasmic expression within the atypical infiltrate representing hairy cell leukemia (magnification ×20). All images were taken using an Olympus BX50 microscope and a SPOT Insight 4 camera and SPOT 5.0 advanced software.

In B-cell neoplasms, *BRAF* V600E mutations are highly sensitive for hairy cell leukemia (HCL), seen in essentially 100% of cases. Arcaini et al. demonstrated this by developing an allele-specific PCR assay which demonstrated that all HCL cases in their study carried this mutation [9]. *BRAF* V600E mutations have also been identified in a subset of marginal zone lymphomas as well as some histiocytic neoplasms [21,22]. Currently, targeted antibodies are commercially available for this mutation [23] and clinically used to support the diagnosis of HCL (Figure 2). Additionally, computational analysis shows that the surrounding peptide region is predicted to be soluble and the peptide is projected to be conjugatable to KLH or BSA. Targeted treatment is available for patients with BRAF mutations and includes drugs such as vemurafenib [24].

### 3.2. RHOA G17V

The *RHOA* gene encodes the RhoA GTPase, which operates as a switch in signal transduction cascades and is primarily involved in actin cytoskeleton organization [25]. The RhoA GTPase is regulated by guanine nucleotide exchange factors (GEFs), which replaces GDP attached to the RhoA GTPase with GTP, and GAPs, which dephosphorylates the attached GTP. The GEFs and GAPs work to activate and inactivate the *RHOA* signal pathways, respectively [10]. The RhoA GTPase is monomeric in structure with a core G domain characterized by a set of five conserved sequence motifs [26]. The first motif in this domain features a glycine at position 17 (G17).

*RHOA* is mutated in numerous hematopoietic malignancies, but its most significant point mutation is seen at G17V which is strongly associated with angioimmunoblastic T-cell lymphoma (AITL), found in approximately 70% of cases and peripheral T-cell lymphomas with follicular helper phenotype [27]. The valine side chain compromises interaction between RhoA and the guanine base in GDP and GTP. As a result, the G17V mutation in *RHOA* inhibits GDP to GTP exchange, which essentially inactivates the RhoA GTPase in a dominant-negative manner. WT RhoA acts as a tumor suppressor while the mutated G17V RhoA loses this activity [10]. Currently, there are no antibodies available for the *RHOA* G17V mutation on the market or from research groups. In our analysis, we found that *RHOA* G17V displayed unfavorable solubility and unfavorable conjugation characteristics of the surrounding peptide region and therefore may not be amenable to antibody production (Table 1).

### 3.3. IRF8 K66R

Interferon (IFN) regulatory factor 8 (*IRF8*) is significantly expressed in plasmacytoid dendritic cells and B-cells and encodes for a transcription factor belonging to the IRF family [28]. Downstream IRF8 activity is induced when type 2 interferon (IFN-γ) binds with its heterodimeric IFN gamma receptor and activates the Janus kinase (JAK)-signal transducer and activator of transcription (STAT) pathway [29]. Once active, IRF8 targets multiple genetic elements including the IFN-stimulated response element (ISRE), the ETs/IRF composite element and the IFN-γ activation site (GAS) [30]. Generally, IRFs help to regulate expression of IFN stimulated genes (ISGs) that mainly contribute to anti-viral defense mechanisms [30]. IRF8 acts both as an activator and repressor and plays a key role in hematopoietic cell development by regulating expression of target genes relevant to myeloid and lymphoid cell differentiation [31]. IRF8 murine knockouts have shown compromised cellular maturation, increased levels of resistance to apoptosis and reduced immune system activity [32,33]. The N-terminal region contains a conserved DNA-binding domain, while the C-terminal region is more variable and responsible for IRF function [34]. The point mutation c.197A>G produces the amino acid substitution p.Lys66Arg (K66R) located in the conserved N-terminal region, specifically impacting loop 2 (C-terminal to alpha helix-2) and is predicted to impact DNA-protein binding [34]. This specific variant is found uniquely associated with pediatric-type follicular lymphoma (PTFL), occurring at relatively high frequencies in two recent studies (15% and 50%, n = 39 and n = 6, respectively) [34]. Antibodies for *IRF8* K66R detection are unavailable commercially, although flanking regions appear to be soluble and the region itself is likely conjugatable to KLH or BSA (Table 1).

### 3.4. MYD88 L265P

The myeloid differentiation primary response 88 (*MYD88*) gene codes for an intracellular adaptor protein that plays a fundamental role in activating the innate immune response [35]. MYD88-dependent pathways include the Toll-like receptor (TLR) and interleukin-1 receptor (IL-1R) pathways [35,36,37]. TLRs are responsible for recognition of pathogen-associated molecular patterns (PAMPs), while IL-1Rs are responsible for recognition of the cytokine, Interleukin-1; both lead to downstream activation of the transcription factors, nuclear factor-kB (NF-kB), a central regulator of the eukaryotic inflammatory and innate immune responses and mitogen-activated protein (MAP) kinase, responsible for activation of cell division [35,38]. Following receptor activation, MYD88 directly associates with the cytoplasmic region of dimerized receptors via its Toll-interleukin-1 receptor (TIR) domain and recruits downstream IL-1R-associated kinase (IRAK) family kinases to form a complex called the Myddosome via interaction of its N-terminus death domain (DD) [39,40]. Under normal environmental conditions, NF-kB is inactive, complexed with inhibitory proteins (IkB) that prevent it from relocating into the nucleus [39]. When released from the IkB complex following IkB enzymatic degradation, NF-kB rapidly relocates and activates transcription of hundreds of genes that lead to multiple immune response functions including proinflammatory cytokine/chemokine secretion, hematopoiesis, cell proliferation, adhesion, migration, and apoptosis [39,41,42,43,44].

The MYD88 substitution, p.Leu265Pro (L265P), is produced by a single missense non-synonymous point mutation c.755T>C within the Toll-interleukin-1 receptor (TIR) domain [45]. The TIR domain is necessary for the proper functioning of MYD88 and is highly conserved, however, L265P occurs in a mutational hotspot relevant to malignant diseases [45,46]. L265P has been hypothesized to promote assembly of MYD88 homodimers or increased affinity to TLR or IL-1R TIR domains which may impact Myddosome assembly and/or signal initiation [35]. Complementation experiments concluded that L265P is a gain-of-function mutation and that mutant isoform NF-kB activation was significantly stronger compared to the wild type isoform, resulting in significant upregulation of NF-kB gene targets [45]. NF-kB mis-regulation and/or constitutive expression has been widely found in human malignancies [47].

*MYD88* L265P is pathogenic, occurring at significant frequencies in hematologic malignancies such as chronic lymphocytic leukemia, marginal zone lymphomas of the ocular adnexa, primary central nervous system large B-cell lymphomas and multiple myeloma amongst numerous others [46,48,49,50,51,52,53]. In three separate studies, L265P was found in over 90% of patients with lymphoplasmacytic lymphoma (LPL) and from 86–100% of patients with related Waldenström macroglobulinemia (WM) [54,55,56]. Additionally, RNA resequencing of activated B-cell (ABC) diffuse large B-cell lymphoma (DLBCL) tumors found 29% were positive for the L265P variant and 56% of the ABC DLBCL cases had an increased copy number of the L265P variant [45]. The strong association of *MYD88* L265P with LPL and WM suggests that this substitution would be useful in distinguishing these diseases from other similar malignancies, however, point mutation specific antibodies are not yet available. Although the peptide region flanking the variant is soluble, conditions for chemical conjugation of the local region flanking the mutation to KLH or BSA may not be favorable (Table 1). In a 3D model of MYD88 the L265P mutation is positioned in a ß-sheet (specifically a beta-beta loop) in the hydrophobic core of the TIR region and not surface accessible in the native protein [12,45].

### 3.5. IDH2 R172K

The *IDH2* gene codes for an enzyme that converts isocitrate to 2-oxoglutarate in the Krebs cycle, but mutations at specific arginine residues at active sites alter the function of the enzyme. The mutated enzymes convert 2-oxoglutarate to 2-hydroxyglutarate (2-HG), resulting in 2-HG quantities up to 100-fold higher than in wild type cells. 2-HG acts as a competitive inhibitor of multiple enzymes that are dependent on 2-oxoglutarate, such as prolyl hydroxylases, which affect hypoxia signaling. Other enzymes that could be affected by overproduction of 2-HG include enzymes involved in histone methylation and DNA methylation. The effects of these mutations in the *IDH2* gene are linked to leukemogenesis and potentially solid cancers [13,57].

The active site arginine at position 172 mutated to a lysine (R172K) leads to excessive 2-HG production. *IDH2* R172K appears to be significantly linked to hematopoietic and lymphoid neoplasms and within that group seen in angioimmunoblastic T-cell lymphoma (AITL) [58]. The *IDH2* R172K mutation is found in up to 45% of AITL patients; it additionally is seen in acute myeloid leukemia (AML) and tends to confer worse prognoses to patients with de novo AML [59]. Antibodies for this mutation are available [60], which supports the findings that the surrounding peptide region is soluble and a 21-mer peptide consisting of the mutation is conjugatable.

Current therapies targeting IDH2 include the use of the inhibitory compound enasidenib, approved for clinical use for the treatment of relapsed or refractory AML, with multiple additional compounds in development. However, enasidenib was designed for, and is most effective against, the *IDH2* R140Q mutation. One inhibitory compound in development, labeled TQ05310, showed significant efficacy in inhibiting *IDH2* R172K as well as *IDH2* R140Q [57].

### 3.6. DNMT3A R882H

As its name implies, DNA (cytosine-5)-methyltransferase 3A (DNMT3A) is a protein that enzymatically catalyzes DNA methylation by transferring methyl groups to CpG structures in DNA. While well known to be mutated in myeloid malignancies, *DNMT3A* is additionally mutated in roughly 30% of nodal T-cell lymphomas [14]. Mutations in *DNMT3A* result in epigenetic abnormalities. It is believed that *DNMT3A* mutations arise in hematopoietic progenitor stem cells, upstream of T-cell lineage commitment based on the observation of identical *DNMT3A* and *TET2* mutations in tumor and normal cells in T-cell lymphoma patients [61]. The most common mutation is a point mutation R882H which involves the catalytic and DNA binding site, but mutations can occur across the entire protein. This change is known to be an inactivating mutation based on in vitro studies [62]. While methylation therapies targeting *DNMT3A*-mutated myeloid neoplasms exist, such targeted therapies are not currently in use for T-cell lymphomas with *DNMT3A* mutations.

There are no point mutation specific antibodies to *DNMT3A* R882H readily available on the market. Computational analysis predicts that a 21-mer peptide region surrounding the mutation would likely be soluble but not amenable to generation of a point mutation specific antibody. The peptide region is predicted to be modifiable for chemical conjugation to BSA or KLH.

### 3.7. KRAS G12D

KRAS belongs to a group of small GTP-binding proteins known as the RAS superfamily or RAS-like GTPases and acts as a so-called “on-off” switch to promote cellular growth and division or maturation. The switch from an inactive to an active form is regulated by intracellular signals. Once the GTP is bound to the KRAS protein, KRAS undergoes conformational changes that involve two regions of the protein, thus activating it [63].

Changes in the shape of the KRAS protein affect its interactions with multiple downstream transducers such as GAPS which amplify the GTPase activity of the RAS protein 100,000-fold [64]. The change also affects interactions with guanine-exchanging/releasing factors (GEFs/GRFs) promoting the release of GTP. The KRAS protein also has intrinsic GTPase activity, stimulated by GAPs, which acts as a timer associated with direct interactions with the effectors [65].

The amino acid positions that account for most of these mutations include G12, G13 and Q61. The different protein isoforms, despite their raw similarity, also behave very differently when expressed in non-native tissue types, likely due to differences in the C-terminal hyper-variable regions. Dysregulation of isoform expression has been shown to be a driver event in carcinogenesis, as well as missense mutations at the three hotspots previously mentioned [66].

While *KRAS* is mutated in many tumors including non-hematopoietic malignancies, T-lymphoblastic lymphoma/leukemia (T-ALL), which accounts for up to 15% of pediatric and 25% of adult ALL cases, may have a *KRAS* G12D mutation. The mutation has been found to be an oncogenic driver of T-ALL [67]. Antibodies for this mutation are commercially available [68]. Computational analysis of the mutational site and flanking regions indicate poor solubility for a peptide generated from this region, and that the peptide region is of low complexity. These parameters would suggest production of a point mutation specific antibody would be difficult.

### 3.8. NRAS Q61K

NRAS belongs to the same family of GTPases as KRAS. NRAS is an oncogene encoding a membrane protein that shuttles between the Golgi apparatus and the plasma membrane. This shuttling is regulated by the ZDHHC9-GOLGA7 complex. The protein that is encoded has an intrinsic GTPase activity which is activated by a GEF and is inactivated by a GTPase activating protein.

Variable isoforms, despite having some similarities, also behave differently when expressed in non-native tissue types, which is thought to be likely due to differences in the C-terminal hyper-variable regions. Interestingly, dysregulation of isoform expression has been shown to be oncogenic. In general, Q61K confers a loss of function on the NRAS protein as indicated by activation of downstream pathway signaling, increased survival, and transformation of cultured cells [69].

Cutaneous T-cell lymphomas (CTCLs) make up about 4% of non-Hodgkin lymphomas and the *NRAS* Q61K mutation in Hut78 cells has been determined as a significant oncogenic factor in CTCL development [16]. There are no targeted antibodies for this mutation currently available, even though the peptide region appears to have favorable solubility and conjugation properties for antibody production from computational analysis. Finally, targeted therapies are available and sorafenib is a RAF kinase-targeting drug and has been shown to block cell growth in CTCL cell lines that harbor this point mutation [16].

### 3.9. SF3B1 K700E

The splicing factor 3 subunit 1 (*SF3B1*) gene encodes for the largest subunit of the splicing factor 3b protein complex. Splicing factor 3b, together with splicing factor 3a and a 12S RNA unit, forms the U2 small nuclear ribonucleoproteins complex [70]. This splicing complex binds precursor mRNA upstream of the intron’s branch site in an independent manner and could anchor the U2 snRNP to the pre-mRNA. Splicing factor 3b is also a component of the minor U12-type spliceosome [70].

Somatic *SF3B1* mutations are found in approximately 30% of patients with myelodysplastic syndrome (MDS) and as many as 80% of patients with the MDS subtype characterized by ring sideroblasts (MDS-RS) [71]. Interestingly, MDS patients with *SF3B1* mutations have been reported to have better overall and event-free survival than their wild type counterpart. These mutations are also present in upwards of 20% of patients with myelodysplastic/myeloproliferative neoplasms (MDS/MPN) and in 5–18% of patients with chronic lymphocytic leukemia (CLL) [17,72].

The mutations affecting *SF3B1* are typically heterozygous point mutations suspected to be functionally deleterious with R625 and K700E described as major mutation hotspots. [73]. Alterations in this gene was found to dysregulate multiple cellular functions, including heme biosynthesis, immune infiltration, DNA damage response, R-loop formation, telomere maintenance, Notch signaling, as well as NF-kB pathways. Modification of such processes suggests that specific point mutations in *SF3B1* play a significant role in oncogenesis within hematolymphoid neoplasms [74].

Currently, there are no antibodies readily available for this mutation, even though the peptide region surrounding the mutation K700E appears to have favorable solubility and is conjugatable. Targeted therapy has been evaluated in preclinical models [75].

### 3.10. ID3 L64F

Inhibitor of DNA binding (ID) proteins have been shown to regulate normal cellular development since these proteins lack a DNA-binding domain and inhibit transcription through the formation of nonfunctional heterodimers with other basic helix-loop-helix (bHLH) proteins [76,77,78]. The *ID3* gene encodes the DNA-binding protein inhibitor ID-3, which is a helix-loop-helix protein involved in negatively regulating the DNA-binding transcription factor TCF3 and behaves as a tumor suppressor [79]. *ID3* is highly expressed in embryonic tissue but expression decreases as cells differentiate [80]. In adult tissues, *ID3* expression is highest in proliferating and undifferentiated cells. Expression of *ID3* is also inducible in response to various stimuli across multiple cell types [79].

The most common mutation is a point mutation resulting in a substitution of a phenylalanine for the wild type leucine at the hotspot L64F. This mutation targets the conserved loop region of ID3, which has been shown to demonstrate loss of the tumor suppressor function of ID3 in in vitro functional studies [81]. These studies performed on mice showed selective defects in humoral immunity and that ID3 is required for B-cell receptor-mediated B-cell proliferation [81].

Genetic alterations of *ID3* in cancers are generally rare, and are most frequently seen in Burkitt lymphoma, and less commonly in various solid tumors as mutations, amplifications, and deletions. In Burkitt lymphoma, mutations involving *ID3* are one of the more common neoplastic genetic alterations, occurring in 40% to 58% of cases [18]. Burkitt lymphoma bearing ID3 alteration tends to have at least two *ID3* mutations, on separate alleles, consistent with the tumor suppressor function of ID3. These *ID3* mutations include nonsense, frameshift, splice donor site, and missense mutations. Mutations in *ID3* have been shown to increase cell cycle progression and the expression of proliferation-associated genes in Burkitt lymphoma [76].

There are no current point mutation specific antibodies available for the L64F variant of *ID3*. Computational modeling and prediction of the probability to generate antibodies against this region show that the peptide region demonstrates unfavorable solubility though it is conjugatable.

Currently, there are no targeted therapeutic targets available for the L64F variant of the *ID3* gene. However, the ability of wild type *ID3* to decrease cell proliferation in Burkitt lymphoma suggests ID3 mimetics may be a potential therapeutic approach in this disease process.

### 3.11. EZH2 Y646H

The epigenetic regulator EZH2 is a subunit of the polycomb repressive complex 2 (PRC2) which methylates histones resulting chromatin compaction and in transcriptional silencing [82,83,84,85]. While *EZH2* is mutated at multiple sites in hematopoietic neoplasms, the Y646 amino acid is recurrently and significantly mutated in 7–22% of follicular lymphoma (FL) cases, with up to 40% of the mutations specifically a Y646H alteration [19]. The mutation is additionally seen in 22% of germinal center DLBCLs [86]. The Y646N amino acid change has been shown in vitro to confer a gain-of-function of enzymatic activity resulting in methylation of lysine 27 of histone H3 to favor trimethylation and suppress expression of polycomb targets [87,88]. Interestingly in mouse models attempting to recapitulate human disease, *EZH2* overexpression in mice leads to myeloproliferative disorders rather than lymphomas [89].

Currently, targeted therapies for EZH2 in FL exist and on 18 June 2020 the U.S. Food and Drug Administration (FDA) granted approval to tazemetostat (Tazverik) which is an EZH2 inhibitor for use in adult patients in selected clinical scenarios of relapsed or refractory FL [90]. While there are currently no targeting antibodies readily available for the Y646 *EZH2* mutation, it nonetheless is a reasonable target for point mutation specific antibodies. However, based on computational modeling and data analysis, the peptide region contains unfavorable conditions for conjugation which may impair production of any such antibodies.

## 4. Discussion

Disease defining genetic mutations are currently a hot topic within medicine and a heavily researched area due to the complimentary role it plays in clinical diagnostics and prognostic stratification. Current modalities to elucidate these mutations have various different bottlenecks in regard to turnover time and costliness of a specific targeted test. Commercially available immunohistochemical antibodies against these mutations have allowed pathologists to render accurate and expeditious results in order to provide data to construct treatment plans and define actionable targets for therapy.

In this review, we examined several key mutations relevant to T-cell and B-cell malignancies that not only are diagnostic but may have prognostic implications. Many of the identified mutations are located in soluble regions of the protein, allowing conjugation, a key requirement for targeted antibody development. Furthermore, the identified mutations have sufficient sequence complexity to permit antibody specificity. While not commercially available at present, the discussed genes and point mutations have great potential for antibody development, expediting diagnosis and offering novel treatment options for hematolymphoid malignancies. Although determining the specificity of an antibody against a target antigen can be challenging, this process is simplified when assessing the impact of a point mutation on antibody specificity. Antibodies can be tested using cell lines or cases of lymphoma with known point mutations and compared to non-mutant negative controls. This technical advantage permits rapid validation of the predicted antibody specificity in vitro which is directly applicable to the development of diagnostic assays for hematologic malignancies.

There are limitations to our interpretation of the predicted possibility of generating targeted antibodies to these point mutations. Our method focused on 21-mers and assumed that these peptides would be structurally similar to the full protein and would chemically permit antibody access to the target residues. Our analysis also weighs the accessibility of the target amino acid by using software that predicts the accessibility of the residue present on the surface. This method is not absolute, as evidenced by the *IDH2* R172K and *KRAS* G12D mutations, which has commercially available point mutation specific antibodies thought our modelling predicted otherwise. Future studies may benefit from the use of 3D protein model prediction software, such as DeepMind’s AlphaFold (https://deepmind.com/research/case-studies/alphafold, accessed on 4 January 2021), to predict the impact of a point mutation on overall protein structure. Additionally, it may be relevant to analyze and predict the dynamic impact of point mutations on a protein structure using molecular dynamic simulations.

Our findings illustrate the immense opportunity for generating diagnostic antibodies against point mutations related to hematologic malignancies. In theory, availability of these antibodies will readily provide a cost-effective and time conserving snapshot assay. Additionally, current NGS techniques have multiple bottlenecks as certain types of tissues are suboptimal for processing due to degradation of DNA (i.e., decalcified bone marrow) or when low volume specimens (fine needle aspirations and core needle biopsies) may produce false negatives as tumor burden may be focal. The use of these antibodies will provide pathologists with alternative modalities of attaining diagnostic results in situations where accessible tissue is suboptimal for genetic studies. Additionally, we believe that these antibodies will provide spatial information in identifying neoplastic clonal populations (i.e., lymphoid versus myeloid, B versus T) that may aid in further delineating morphologically complex cases.

In summary, further development of this diagnostic strategy has the potential to accelerate diagnosis, provide spatial information of neoplastic clones and guide treatment for a diverse range of hematological cancers.

## Figures and Tables

**Table 1:** Characteristics of genes and proteins with point mutations relevant to B and T cell malignancies.

Protein	Mutation	Disease Prevalence	Commercial Antibody Available	Predicted Solubility	Conjugation Chemistry Likelihood			AA Sequence Complexity	Surface Epitope Exposure 0–9; 9 Most Accessible (PDB File Code)	
					MBS	EDC	aEDC		Wild-Type	Mutant
BRAF	V600E	~100% of HCL [9]	Yes	Likely	Yes	No	No	High	8 (3Q4C)	5 (4MNF)
RHOA	G17V	Up to 70% of AITL [10]	No	Unlikely	No	No	No	High	2 (1FTN)	NA
IRF8	K66R	~15% of PTFL [11]	No	Likely	Yes	No	No	High	NA **	NA **
MYD88	L265P	>90% of LPL [12]	No	Likely	No	No	No	High	NA	0 (4EO7)
IDH2	R172K	up to 45% of AITL [13]	Yes	Likely	Yes	No	No	High	2 (5SVO)	0 (5SVN)
DNMT3A	R882H	~30% of nodal TCL [14]	No	Likely	Yes	No	Yes	High	6 (4U7P)	2 (6W89)
KRAS	G12D	~10–15% of B and T ALL [15]	Yes	Unlikely	Yes	No	No	Low	9 (4EPT)	5 (6GJ7)
NRAS	Q61K	up to 11% of CTCL [16]	No	Likely	Yes *	No	No	High	2 (5UHV)	NA
SF3B1	K700E	up to 18% of CLL [17]	No	Likely	Yes	No	Yes	High	2 (5IFE)	NA
ID3	L64F	Up to 58% of BL [18]	No	Unlikely	Yes	No	Yes	High	1 (2LFH)	NA
EZH2	Y646H	~9% of FL [19]	No	Likely	No	No	No	High	0 (4MI5)	NA

AITL = angioimmunoblastic T-cell lymphoma; HCL = Hairy cell leukemia; PTFL = Pediatric-type Follicular Lymphoma; CTCL = Cutaneous T-cell lymphoma; ALL = Acute Lymphoblastic Leukemia; CLL = Chronic Lymphocytic Leukemia; BL = Burkitt’s lymphoma; FL = Follicular lymphoma. * The 21-mer used for the NRAS mutation contains a cysteine residue at the N-terminus, which is predicted to be conjugatable with MBS. MBS = m-Maleimidobenzoyl-N-hydroxysuccinimide ester; EDC = 1-ethyl-3-[3-dimethylaminopropyl]carbodiimide hydrochloride; aEDC = activated EDC. ** The surface epitope exposure for IRF8 is unknown due the unavailability of a 3D structure from RCSB PDB.

## References

[1] The 2016 Revision to the World Health Organization Classification of Myeloid Neoplasms and Acute Leukemia | Blood | American Society of Hematology. https://ashpublications.org/blood/article/127/20/2391/35255/The-2016-revision-to-the-World-Health-Organization.

[2] Feller J.K., Yang S., Mahalingam M. (2013). Immunohistochemistry with a mutation-specific monoclonal antibody as a screening tool for the BRAFV600E mutational status in primary cutaneous malignant melanoma. Mod. Pathol..

[3] Yu J., Kane S., Wu J., Benedettini E., Li D., Reeves C., Innocenti G., Wetzel R., Crosby K., Becker A. (2009). Mutation-Specific Antibodies for the Detection of EGFR Mutations in Non–Small-Cell Lung Cancer. Clin. Cancer Res..

[4] Disanto M.G., Ambrosio M.R., Rocca B.J., Ibrahim H.A.H., Leoncini L., Naresh K.N. (2016). Optimal Minimal Panels of Immunohistochemistry for Diagnosis of B-Cell Lymphoma for Application in Countries With Limited Resources and for Triaging Cases Before Referral to Specialist Centers. Am. J. Clin. Pathol..

[5] Lear S., Cobb S.L. (2016). Pep-Calc.com: A set of web utilities for the calculation of peptide and peptoid properties and automatic mass spectral peak assignment. J. Comput. Aided Mol. Des..

[6] Letunic I., Khedkar S., Bork P. (2021). SMART: Recent updates, new developments and status in 2020. Nucleic Acids Res..

[7] Wootton J.C., Federhen S. (1993). Statistics of local complexity in amino acid sequences and sequence databases. Comput. Chem..

[8] Porollo A.A., Adamczak R., Meller J. (2004). POLYVIEW: A flexible visualization tool for structural and functional annotations of proteins. Bioinformatics.

[9] Arcaini L., Zibellini S., Boveri E., Riboni R., Rattotti S., Varettoni M., Guerrera M.L., Lucioni M., Tenore A., Merli M. (2012). The BRAF V600E mutation in hairy cell leukemia and other mature B-cell neoplasms. Blood.

[10] Chiba S., Enami T., Ogawa S., Sakata-Yanagimoto M. (2015). G17V RHOA: Genetic evidence of GTP-unbound RHOA playing a role in tumorigenesis in T cells. Small GTPases.

[11] Schmidt J., Ramis-Zaldivar J.E., Nadeu F., Gonzalez-Farre B., Navarro A., Egan C., Montes-Mojarro I.A., Marafioti T., Cabeçadas J., van der Walt J. (2017). Mutations of MAP2K1 are frequent in pediatric-type follicular lymphoma and result in ERK pathway activation. Blood.

[12] Rossi D. (2014). Role of MYD88 in lymphoplasmacytic lymphoma diagnosis and pathogenesis. Hematology.

[13] Cairns R.A., Iqbal J., Lemonnier F., Kucuk C., de Leval L., Jais J.-P., Parrens M., Martin A., Xerri L., Brousset P. (2012). IDH2 mutations are frequent in angioimmunoblastic T-cell lymphoma. Blood.

[14] Yang L., Rau R., Goodell M.A. (2015). DNMT3A in haematological malignancies. Nat. Rev. Cancer.

[15] Kindler T., Cornejo M.G., Scholl C., Liu J., Leeman D.S., Haydu J.E., Fröhling S., Lee B.H., Gilliland D.G. (2008). K-RasG12D–induced T-cell lymphoblastic lymphoma/leukemias harbor Notch1 mutations and are sensitive to γ-secretase inhibitors. Blood.

[16] KieΔling M.K., Nicolay J.P., Schlör T., Klemke C.-D., Süss D., Krammer P.H., Gülow K. (2017). NRAS mutations in cutaneous T cell lymphoma (CTCL) sensitize tumors towards treatment with the multikinase inhibitor Sorafenib. Oncotarget.

[17] Wan Y., Wu C.J. (2013). SF3B1 mutations in chronic lymphocytic leukemia. Blood.

[18] Schmitz R., Young R.M., Ceribelli M., Jhavar S., Xiao W., Zhang M., Wright G., Shaffer A.L., Hodson D.J., Buras E. (2012). Burkitt Lymphoma Pathogenesis and Therapeutic Targets from Structural and Functional Genomics. Nature.

[19] Bödör C., O’Riain C., Wrench D., Matthews J., Iyengar S., Tayyib H., Calaminici M., Clear A., Iqbal S., Quentmeier H. (2011). EZH2 Y641 mutations in follicular lymphoma. Leukemia.

[20] Ritterhouse L.L., Barletta J.A. (2015). BRAF V600E mutation-specific antibody: A review. Semin. Diagn. Pathol..

[21] Pillonel V., Juskevicius D., Ng C.K.Y., Bodmer A., Zettl A., Jucker D., Dirnhofer S., Tzankov A. (2018). High-throughput sequencing of nodal marginal zone lymphomas identifies recurrent BRAF mutations. Leukemia.

[22] Tadmor T., Tiacci E., Falini B., Polliack A. (2012). The BRAF-V600E mutation in hematological malignancies: A new player in hairy cell leukemia and Langerhans cell histiocytosis. Leuk. Lymphoma.

[23] Anti-BRAF (V600E) Antibody Produced in Rabbit SAB4200772. Sigma-Aldrich. https://www.sigmaaldrich.com/catalog/product/sigma/sab4200772.

[24] Dietrich S., Pircher A., Endris V., Peyrade F., Wendtner C.-M., Follows G.A., Hüllein J., Jethwa A., Ellert E., Walther T. (2016). BRAF inhibition in hairy cell leukemia with low-dose vemurafenib. Blood.

[25] RHOA ras Homolog Family Member A [Homo Sapiens (Human)]-Gene-NCBI. https://www.ncbi.nlm.nih.gov/gene/387.

[26] Schaefer A., Reinhard N.R., Hordijk P.L. (2014). Toward understanding RhoGTPase specificity: Structure, function and local activation. Small GTPases.

[27] Watatani Y., Sato Y., Miyoshi H., Sakamoto K., Nishida K., Gion Y., Nagata Y., Shiraishi Y., Chiba K., Tanaka H. (2019). Molecular heterogeneity in peripheral T-cell lymphoma, not otherwise specified revealed by comprehensive genetic profiling. Leukemia.

[28] Blood Atlas—IRF8—The Human Protein Atlas. https://www.proteinatlas.org/ENSG00000140968-IRF8/blood.

[29] Tamura T., Ozato K. (2002). Review: ICSBP/IRF-8: Its Regulatory Roles in the Development of Myeloid Cells. J. Interferon Cytokine Res..

[30] Chen K., Liu J., Cao X. (2017). Regulation of type I interferon signaling in immunity and inflammation: A comprehensive review. J. Autoimmun..

[31] Tamura T., Thotakura P., Tanaka T.S., Ko M.S.H., Ozato K. (2005). Identification of target genes and a unique cis element regulated by IRF-8 in developing macrophages. Blood.

[32] Yang J., Hu X., Zimmerman M., Torres C.M., Yang D., Smith S.B., Liu K. (2011). Cutting Edge: IRF8 Regulates Bax Transcription In Vivo in Primary Myeloid Cells. J. Immunol..

[33] IRF8 Regulates Transcription of Naips for NLRC4 Inflammasome Activation: Cell. https://www.cell.com/cell/fulltext/S0092-8674(18)30232-0?_returnURL=https%3A%2F%2Flinkinghub.elsevier.com%2Fretrieve%2Fpii%2FS0092867418302320%3Fshowall%3Dtrue.

[34] Ozawa M.G., Bhaduri A., Chisholm K.M., Baker S.A., Ma L., Zehnder J.L., Luna-Fineman S., Link M.P., Merker J.D., Arber D.A. (2016). A study of the mutational landscape of pediatric-type follicular lymphoma and pediatric nodal marginal zone lymphoma. Mod. Pathol..

[35] Deguine J., Barton G.M. (2014). MyD88: A central player in innate immune signaling. F1000Prime Rep..

[36] Akira S., Hoshino K. (2003). Myeloid Differentiation Factor 88–Dependent and –Independent Pathways in Toll-Like Receptor Signaling. J. Infect. Dis..

[37] Kawai T., Sato S., Ishii K.J., Coban C., Hemmi H., Yamamoto M., Terai K., Matsuda M., Inoue J., Uematsu S. (2004). Interferon-alpha induction through Toll-like receptors involves a direct interaction of IRF7 with MyD88 and TRAF6. Nat. Immunol..

[38] Gilmore T.D. (1999). The Rel/NF-κB signal transduction pathway: Introduction. Oncogene.

[39] Kramer I.M., Kramer I.M. (2016). Chapter 13—Activation of the Innate Immune System: The Toll-Like Receptor-4 and Signaling through Ubiquitinylation. Signal Transduction.

[40] Lin S.-C., Lo Y.-C., Wu H. (2010). Helical assembly in the MyD88–IRAK4–IRAK2 complex in TLR/IL-1R signalling. Nat. Cell Biol..

[41] Dolcet X., Llobet D., Pallares J., Matias-Guiu X. (2005). NF-kB in development and progression of human cancer. Virchows Archiv..

[42] Pahl H.L. (1999). Activators and target genes of Rel/NF-kappaB transcription factors. Oncogene.

[43] May M.J., Ghosh S. (1998). Signal transduction through NF-κB. Immunol. Today.

[44] Sims J.E., March C.J., Cosman D., Widmer M.B., MacDonald H.R., McMahan C.J., Grubin C.E., Wignall J.M., Jackson J.L., Call S.M. (1988). cDNA expression cloning of the IL-1 receptor, a member of the immunoglobulin superfamily. Science.

[45] Ngo V.N., Young R.M., Schmitz R., Jhavar S., Xiao W., Lim K.-H., Kohlhammer H., Xu (2011). W.; Yang, Y.; Zhao, H.; et al. Oncogenically active MYD88 mutations in human lymphoma. Nature.

[46] Chang M.T., Asthana S., Gao S.P., Lee B.H., Chapman J.S., Kandoth C., Gao J., Socci N.D., Solit D.B., Olshen A.B. (2016). Identifying recurrent mutations in cancer reveals widespread lineage diversity and mutational specificity. Nat. Biotechnol..

[47] Vlahopoulos S.A. (2017). Aberrant control of NF-κB in cancer permits transcriptional and phenotypic plasticity, to curtail dependence on host tissue: Molecular mode. Cancer Biol. Med..

[48] Munshi M., Liu X., Chen J.G., Xu L., Tsakmaklis N., Demos M.G., Kofides A., Guerrera M.L., Jimenez C., Chan G.G. (2020). SYK is activated by mutated MYD88 and drives pro-survival signaling in MYD88 driven B-cell lymphomas. Blood Cancer J..

[49] Chen Z., Zou Y., Liu W., Guan P., Tao Q., Xiang C., Zhang W., Ye Y., Yan J., Zhao S. (2020). Morphologic Patterns and the Correlation with MYD88 L265P, CD79B Mutations in Primary Adrenal Diffuse Large B-Cell Lymphoma. Am. J. Surg. Pathol..

[50] Pan S.-T., Wang R.C., Kuo C.-C., Hsieh Y.-C., Su Y.-Z., Chuang S.-S. (2019). MYD88 L265P mutation analysis is a useful diagnostic adjunct for lymphoplasmacytic lymphoma with pleural effusion. Pathol. Int..

[51] Wu Y.-Y., Jia M.-N., Cai H., Qiu Y., Zhou D.-B., Li J., Cao X.-X. (2020). Detection of the MYD88L265P and CXCR4S338X mutations by cell-free DNA in Waldenström macroglobulinemia. Ann. Hematol..

[52] Shin D.W., Kim S.-M., Kim J.-A., Park H.S., Hwang S.M., Im K., Kim S., Kim J., Kwon S., Yoon S.S. (2019). Characteristics of Waldenström Macroglobulinemia in Korean Patients According to Mutational Status of MYD88 and CXCR4: Analysis Using Ultra-Deep Sequencing. Clin. Lymphoma Myeloma Leuk..

[53] Lauw M.I.S., Lucas C.-H.G., Ohgami R.S., Wen K.W. (2020). Primary Central Nervous System Lymphomas: A Diagnostic Overview of Key Histomorphologic, Immunophenotypic, and Genetic Features. Diagnostics.

[54] Treon S.P., Xu L., Yang G., Zhou Y., Liu X., Cao Y., Sheehy P., Manning R.J., Patterson C.J., Tripsas C. (2012). MYD88 L265P Somatic Mutation in Waldenström’s Macroglobulinemia. N. Engl. J. Med..

[55] Jiménez C., Sebastián E., Chillón M.C., Giraldo P., Mariano Hernández J., Escalante F., González-López T.J., Aguilera C., de Coca A.G., Murillo I. (2013). MYD88 L265P is a marker highly characteristic of, but not restricted to, Waldenström’s macroglobulinemia. Leukemia.

[56] Varettoni M., Arcaini L., Zibellini S., Boveri E., Rattotti S., Riboni R., Corso A., Orlandi E., Bonfichi M., Gotti M. (2013). Prevalence and clinical significance of the MYD88 (L265P) somatic mutation in Waldenström’s macroglobulinemia and related lymphoid neoplasms. Blood.

[57] Gao M., Zhu H., Fu L., Li Y., Bao X., Fu H., Quan H., Wang L., Lou L. (2019). Pharmacological characterization of TQ 05310, a potent inhibitor of isocitrate dehydrogenase 2 R140Q and R172K mutants. Cancer Sci..

[58] Mutation Overview Page IDH2-p.R172K (Substitution-Missense). https://cancer.sanger.ac.uk/cosmic/mutation/overview?id=173874954.

[59] Mondesir J., Willekens C., Touat M., de Botton S. (2016). IDH1 and IDH2 mutations as novel therapeutic targets: Current perspectives. J. Blood Med..

[60] Anti-IDH2-R172K (Human) mAb (Monoclonal Antibody). https://www.mblintl.com/products/d328-3/.

[61] Sakata-Yanagimoto M., Nakamoto-Matsubara R., Komori D., Nguyen T.B., Hattori K., Nanmoku T., Kato T., Kurita N., Yokoyama Y., Obara N. (2017). Detection of the circulating tumor DNAs in angioimmunoblastic T- cell lymphoma. Ann. Hematol..

[62] Gowher H., Loutchanwoot P., Vorobjeva O., Handa V., Jurkowska R.Z., Jurkowski T.P., Jeltsch A. (2006). Mutational Analysis of the Catalytic Domain of the Murine Dnmt3a DNA-(cytosine C5)-methyltransferase. J. Mol. Biol..

[63] Jančík S., Drábek J., Radzioch D., Hajdúch M. (2010). Clinical Relevance of KRAS in Human Cancers. J. Biomed. Biotechnol..

[64] Gideon P., John J., Frech M., Lautwein A., Clark R., Scheffler J., Wittinghofer A. (1992). Mutational and kinetic analyses of the GTPase-activating protein (GAP)-p21 interaction: The C-terminal domain of GAP is not sufficient for full activity. Mol. Cell. Biol..

[65] Giglione C., Parrini M., Baouz S., Bernardi A., Parmeggiani A. (1997). A new function of p120-GTPase-activating protein—Prevention of the guanine nucleotide exchange factor-stimulated nucleotide exchange on the active form of Ha-Ras p21. J. Biol. Chem..

[66] Prior I.A., Lewis P.D., Mattos C. (2012). A Comprehensive Survey of Ras Mutations in Cancer. Cancer Res..

[67] Kong G., Du J., Liu Y., Meline B., Chang Y.-I., Ranheim E.A., Wang J., Zhang J. (2013). *Notch1* Gene Mutations Target KRAS G12D-expressing CD8^+^ Cells and Contribute to Their Leukemogenic Transformation. J. Biol. Chem..

[68] Anti-Ras (Mutated G12D) Antibody (ab221163)|Abcam. https://www.abcam.com/ras-mutated-g12d-antibody-ab221163.html.

[69] Li A., Ma Y., Jin M., Mason S., Mort R.L., Blyth K., Larue L., Sansom O.J., Machesky L.M. (2012). Activated Mutant NRasQ61K Drives Aberrant Melanocyte Signaling, Survival, and Invasiveness via a Rac1-Dependent Mechanism. J. Investig. Dermatol..

[70] SF3B1 Splicing Factor 3b Subunit 1 [Homo Sapiens (Human)]-Gene-NCBI. https://www.ncbi.nlm.nih.gov/gene/23451.

[71] Papaemmanuil E., Cazzola M., Boultwood J., Malcovati L., Vyas P., Bowen D., Pellagatti A., Wainscoat J.S., Hellstrom-Lindberg E., Gambacorti-Passerini C. (2011). Chronic Myeloid Disorders Working Group of the International Cancer Genome Consortium. SomaticSF3B1Mutation in Myelodysplasia with Ring Sideroblasts. N. Engl. J. Med..

[72] Cazzola M., Della Porta M.G., Malcovati L. (2013). The genetic basis of myelodysplasia and its clinical relevance. Blood.

[73] Zhang J., Ali A.M., Lieu Y.K., Liu Z., Gao J., Rabadan R., Raza A., Mukherjee S., Manley J.L. (2019). Disease-Causing Mutations in SF3B1 Alter Splicing by Disrupting Interaction with SUGP1. Mol. Cell.

[74] Zhou Z., Gong Q., Wang Y., Li M., Wang L., Ding H., Li P. (2020). The biological function and clinical significance of SF3B1 mutations in cancer. Biomark. Res..

[75] Ebert B. Targeting SF3B1 for the Treatment of MDS. https://grantome.com/grant/NIH/P50-CA206963-01A1-5029.

[76] Love C., Sun Z., Jima D., Li G., Zhang J., Miles R., Richards K.L., Dunphy C.H., Choi W.W., Srivastava G. (2012). The genetic landscape of mutations in Burkitt lymphoma. Nat. Genet..

[77] Lasorella A., Benezra R., Iavarone A. (2014). The ID proteins: Master regulators of cancer stem cells and tumour aggressiveness. Nat. Rev. Cancer.

[78] Deed R.W., Hirose T., Mitchell E.L., Santibanez-Koref M.F., Norton J.D. (1994). Structural organisation and chromosomal mapping of the human Id-3 gene. Gene.

[79] Lim R.W.-S., Wu J.-M. (2005). Molecular mechanisms regulating expression and function of transcription regulator “inhibitor of differentiation 3”. Acta Pharmacol. Sin..

[80] Lyden D., Young A.Z., Zagzag D., Yan W., Gerald W., O’Reilly R., Bader B.L., Hynes R.O., Zhuang Y., Manova K. (1999). Id1 and Id3 are required for neurogenesis, angiogenesis and vascularization of tumour xenografts. Nat. Cell Biol..

[81] Pan L., Sato S., Frederick J.P., Sun X.H., Zhuang Y. (1999). Impaired Immune Responses and B-Cell Proliferation in Mice Lacking the Id3 Gene. Mol. Cell. Biol..

[82] Müller J., Hart C.M., Francis N.J., Vargas M.L., Sengupta A., Wild B., Miller E.L., O’Connor M.B., Kingston R.E., Simon J.A. (2002). Histone Methyltransferase Activity of a Drosophila Polycomb Group Repressor Complex. Cell.

[83] Czermin B., Melfi R., McCabe D., Seitz V., Imhof A., Pirrotta V. (2002). Drosophila Enhancer of Zeste/ESC Complexes Have a Histone H3 Methyltransferase Activity that Marks Chromosomal Polycomb Sites. Cell.

[84] Margueron R., Reinberg D. (2011). The Polycomb complex PRC2 and its mark in life. Nat. Cell Biol..

[85] Di Croce L., Helin K. (2013). Transcriptional regulation by Polycomb group proteins. Nat. Struct. Mol. Biol..

[86] Morin R.D., Johnson N.A., Severson T.M., Mungall A.J., An J., Goya R., Paul J.E., Boyle M., Woolcock B.W., Kuchenbauer F. (2010). Somatic mutations altering EZH2 (Tyr641) in follicular and diffuse large B-cell lymphomas of germinal-center origin. Nat. Genet..

[87] Yap D.B., Chu J., Berg T., Schapira M., Cheng S.-W.G., Moradian A., Morin R.D., Mungall A.J., Meissner B., Boyle M. (2011). Somatic mutations at EZH2 Y641 act dominantly through a mechanism of selectively altered PRC2 catalytic activity, to increase H3K27 trimethylation. Blood.

[88] Sneeringer C.J., Scott M.P., Kuntz K.W., Knutson S.K., Pollock R.M., Richon V.M., Copeland R.A. (2010). Coordinated activities of wild-type plus mutant EZH2 drive tumor-associated hypertrimethylation of lysine 27 on histone H3 (H3K27) in human B-cell lymphomas. Proc. Natl. Acad. Sci. USA.

[89] Lund K., Adams P.D., Copland M. (2014). EZH2 in normal and malignant hematopoiesis. Leukemia.

[90] Garber K. (2020). Histone-writer cancer drugs enter center stage. Nat. Biotechnol..

